# Semi-automated genomic newborn screening highlights complexities in reporting

**DOI:** 10.1038/s41525-026-00553-4

**Published:** 2026-02-10

**Authors:** Ayesha Chowdhury, Shashikanth Marri, Lucy Anastasi, Alex Ashenden, Tomas Rozek, Jinghua Feng, Lucas DeJong, Rosalie Kenyon, Dominik Kaczorowski, Hung Nguyen, Khoa Lam, Kirsty Stallard, Tracy Merlin, Enzo Ranieri, Sunita De Sousa, Nicholas Smith, Abhi Kulkarni, Benjamin Saxon, Drago Bratkovic, Christopher Barnett, Carol Wai-Kwan Siu, Hamish S. Scott, Jovanka King, Karin S. Kassahn

**Affiliations:** 1 Department of Genetics and Molecular Pathology, SA Pathology, Adelaide, SA Australia; 2 South Australia Neonatal Screening Centre, Genetics and Molecular Pathology Directorate, SA Pathology at Women’s & Children’s Hospital, North Adelaide, Australia; 3 Adelaide Medical School, Faculty of Health and Medical Sciences, The University of Adelaide, Adelaide, SA Australia; 4 Paediatric and Reproductive Genetics Unit, Women’s and Children’s Health Network, Adelaide, SA Australia; 5 Adelaide Health Technology Assessment (AHTA), School of Public Health, The University of Adelaide, Adelaide, SA 5000 Australia; 6 Sydney Children’s Hospital at Westmead, Sydney, NSW Australia; 7 Endocrinology and Genetics at the Royal Adelaide Hospital, Adelaide, SA Australia; 8 Neurology and Clinical Neurophysiology, Women’s and Children’s Health Network, Adelaide, SA Australia; 9 Paediatric Haematology and Oncology, Women’s and Children’s Health Network, Adelaide, SA Australia; 10 Metabolic Clinic, Women’s and Children’s Health Network, Adelaide, SA Australia; 11 Centre for Cancer Biology, An alliance between SA Pathology and the University of South Australia, Adelaide, SA Australia; 12 Immunology Directorate, SA Pathology, Adelaide, SA Australia; 13 Department of Allergy and Clinical Immunology, Women’s and Children’s Hospital, Adelaide, SA Australia

**Keywords:** Computational biology and bioinformatics, Genetics, Medical research

## Abstract

Newborn screening programs are instrumental in the early detection of treatable conditions in the first days of life. By integrating genomic approaches, there is potential to expand the range of conditions included in these programs. As part of a research study, NewbornsInSA, we validated a genomic newborn screening workflow. Analysis of whole-genome sequencing data was restricted to a virtual panel of 613 genes, selected through engagement with local clinical teams. We assessed the workflow’s performance using retrospective samples with known variant status. To reduce manual curation time, bioinformatics scripts were developed to auto-classify cases into those with no findings and those requiring manual review. We report on early findings from applying this workflow to a prospectively recruited cohort in which five reportable findings have been made to date. We discuss reporting challenges encountered in genes associated with multiple conditions, with incomplete penetrance, or variants associated with only mild phenotypes.

## Introduction

Standard newborn bloodspot screening (stNBS) using tandem mass spectrometry (MS/MS) and dried blood spots (DBS) are highly successful and trusted public health programs, with a near universal uptake^1^. At present, more than 30 conditions are routinely screened for by stNBS in Australia. Recently, with genomic technologies transforming rare disease diagnostics, there has been growing interest in exploring their use in screening applications, including genomic newborn screening (gNBS)^2–4^.

Advances in next-generation sequencing (NGS) technologies, such as whole-genome sequencing (WGS), whole-exome sequencing (WES) and targeted gene panels, have dramatically reduced costs and turnaround times of molecular genetic testing, enabling prompt molecular diagnosis^5,6^. WGS can be performed on DNA derived from DBS and has been explored as a newborn screening tool in recent years^1^. Sequencing data can be filtered to a pre-curated virtual gene panel, restricting variant analysis to conditions appropriate for reporting in a newborn screening context^5,7,8^. gNBS has great potential as a single assay for population-level screening of a range of treatable, early-onset conditions including rare diseases that lack known biochemical markers^1,9,10^. Furthermore, a gNBS virtual gene panel run on a WGS backbone can be readily expanded with minimal additional cost or validation, and with limited impact on laboratory workflows^5^. As the DBS is collected shortly after birth, sequencing can be performed almost immediately to screen for conditions before clinical symptoms develop^5^. Lifetime storage of genomic data may prove a valuable asset as the data can be re-interrogated later in life to answer different clinical questions and fast-track clinical care at any time-point^5^.

For clinical implementation, understanding the false positive and false negative rate of a gNBS test is important^2,10^. One limitation of short-read NGS data, for example, involves repeat sequences or high homology regions, which can reduce the sensitivity and accuracy of variant calling^11,12^. Other technical challenges include complex variant types, such as inversions, which can be difficult to detect using standard bioinformatic approaches. Finally, variant interpretation and classification rely on published scientific evidence, which may or may not be available, thus limiting the recognition of pathogenic variants. And as gNBS is commonly performed as singleton testing, phasing of variants associated with autosomal recessive conditions is typically not available.

As part of the Newborn Screening Model using Integrated multi-omics in South Australia (NewbornsInSA) research study, we validated and implemented a semi-automated whole genome sequencing and analysis workflow and report here on early findings.

## Results

### NewbornsInSA gene list

As a starting point and through the Genomic Screening Consortium for Australian Newborns (GenSCAN)^13^, the 605 gene-condition list developed by the BabyScreen+ study^14,15^ was circulated to South Australian clinical experts. Local teams had the opportunity to add or remove conditions to meet the requirements of their health service, with respect to the benefits of early detection and the ability to provide follow-up clinical care. Proposed changes to the gene panel were discussed in a multi-disciplinary team (MDT) meeting with representatives of relevant medical subspecialties and laboratory specialists. Conditions proposed for inclusion were reviewed against the guiding principles developed by The Generation Study^16^ in particular early disease-onset, the availability of a treatment or intervention that makes a significant difference to health outcomes, and the laboratory’s ability to reliably detect and predict the clinical significance of genetic variants.

All 605 genes were supported for inclusion as part of the NewbornsInSA study. Local clinical teams proposed 25 additional genes and conditions (Table 1, Supplementary Table 1). After detailed review, the following eight genes were approved for inclusion in the final NewbornsInSA virtual gene panel as ‘secondary genes’: *APOB, HNF1B, SLC12A3, CYP1B1, TNNT2, F8, SCN5A*, and *SGSH* (Table 1). Although *APOB* pathogenic variants have incomplete penetrance, treatment can still be initiated early to reduce the risk of cardiovascular events^17,18^. *HNF1B*-related Renal Cysts and Diabetes Syndrome and *SLC12A3*-related Gitelman Syndrome typically present from late childhood to early adulthood; however, earlier presentation may occur with renal cysts and diabetes in individuals with *HNF1B* variants and salt wasting in individuals with *SLC12A3* variants^19–21^. Not all clinically significant *F8* variants, such as intron 22 and intron 1 inversions, can be reliably detected by standard WGS pipelines, but SNVs and indels that are responsible for over 50% of Haemophilia A cases can be reliably detected^22^. Following MDT discussion, these conditions were supported for inclusion with sufficient perceived benefit.

**Table 1:** Description of the 8 ‘secondary’ genes selected for inclusion in the NewbornsInSA gNBS study

Secondary gene	Condition	Pros	Cons
***APOB***	Familial hypercholesterolemia (MIM# 144010) (AD)	Identifying newborns before the onset of familial hypercholesterolemia provides an opportunity to initiate diet and lifestyle modifications and monitor lipid levels from an early age. Statin therapy can be initiated from 8 years of age^17^.	A high chance result for an *APOB* mutation will not provide immediate benefit to the newborn as treatment is initiated only from 8 years of age.
***HNF1B***	Maturity-Onset Diabetes of the Young (MODY5) (MIM# 137920) (AD)	Pre-symptomatic identification of MODY5 may permit early identification of diabetes and commencement of insulin therapy as well as surveillance for renal developmental abnormalities (such as renal cysts) which present in most individuals with pathogenic *HNF1B* variants^20^.	Diagnosis may be challenging due to incomplete penetrance and high phenotypic variability among and between families^21^.
***SLC12A3***	Gitelman syndrome (GS) (MIM# 263800) (AR)	Early detection of GS cases earlier in life reduces morbidity by allowing correction of electrolyte abnormalities, helping to avoid chronic renal insufficiency, chondrocalcinosis, tetany, seizures, or even life-threatening complications such as ventricular arrhythmia^19^.	Variable age of onset – most patients experience onset in adulthood, but some may present in childhood.
***CYP1B1***	Primary congenital glaucoma 3 A (PCG) (MIM# 231300) (AR)	Most PCG cases present in the first year of life^34^. Early initiation of surgical treatments and medical therapies can prevent permanent vision loss^34^. Delaying treatment can lead to significant ocular comorbidities.	*CYP1B1* mutations show variable expressivity between families^35^.
***TNNT2***	Dilated Cardiomyopathy (MIM# 601494) (AD) Familial hypertrophic cardiomyopathy (MIM# 115195) (AD)	These conditions are highly penetrant so babies could benefit from having ongoing monitoring such as echocardiograms every three years^36^.	Variable age of onset^36^.
***F8***	Haemophilia A (MIM# 306700) (XLR)	Most severe cases including those with inversions in intron 22 are from known kindreds which make up 1/3 of new cases in South Australia. However, inclusion of *F8* has a significant impact for the minority of patients affected by point mutations.	Inversions in introns 1 and 22 are difficult to detect with short read sequencing and would require specialised bioinformatics.
***SCN5A***	Long QT syndrome 3 (MIM# 603830) (AD) Brugada syndrome 1 (MIM# 601144) (AD)	Strong actionability in paediatric patients by ClinGen.	Long QT syndrome generally has onset of symptoms in adolescence^37^. Brugada syndrome typically presents in adulthood^38^.
***SGSH***	Mucopolysaccharidosis type IIIA (Sanfilippo A) (MIM# 252900) (AR)	Although in translational phase, therapies for MPSIIIA are equivalent in efficacy and access to those for MPSIIIB^39^. Interventional clinical trials demonstrate that early, presymptomatic treatment ( < 3 y/o) is required to achieve normal neurocognitive developmental trajectory^40^.	Therapies are still in translational phase.

The remaining 18 conditions were not deemed to meet the criteria for inclusion, typically due to the lack of an effective treatment, a variable or later onset in life, or limited clinical validity for predicting the likelihood of disease onset (Supplementary Table 1).

Through focus groups, the gene-condition list was shared with representatives from the general public. The list was further circulated among patient advocacy groups who are partners in the NewbornsInSA study. Based on stakeholder feedback, and to simplify communication with members of the public, the gene-condition list was grouped into fourteen disease categories. Each category included a brief description of the affected body system: audiology, cardiology, dermatology, endocrinology, gastroenterology, hematology, immunology, metabolic, nephrology, neurology, oncology, ophthalmology, respiratory, and multi-system disorders. The final curated gene-condition list is available on the NewbornsInSA website for parents and research participants to access^23^.

### Validation of the gNBS workflow using retrospective DBS samples

To assess the specificity and sensitivity of the NewbornsInSA gNBS workflow, 46 DBS cards of patients with prior diagnostic investigations were selected. These included 31 positive diagnoses spanning a range of condition types and modes of inheritance (autosomal dominant, autosomal recessive, and X-linked), 2 positive diagnoses in genes not included for reporting in the NewbornsInSA study, 3 cases with carrier status for autosomal recessive conditions, 5 cases with variants of uncertain significance (VUS) and 5 negative cases. For each case selected in this validation study, the corresponding DBS card was retrieved from archive in the newborn screening laboratory and sequenced using WGS. Of the 46 retrospective samples, 44 met the desired sequencing yield of 100 Gb with a resulting ~ 28-30x mean coverage. Two samples achieved only 22–23x mean coverage, and in a prospective setting, would require top-up sequencing to meet the desired sequence yield (Supplementary Table 2). Base Q30 for all samples exceeded the vendor-specified threshold of >85%. We observed a read duplication rate between 10 and 15% in these retrospective DBS samples, however, overall sequence yield remained sufficient to meet the target sequence coverage, even after removing PCR duplicates.

### Results from blinded gNBS analysis

Variant annotation and prioritisation were performed using a dual strategy, with the Illumina Emedgene v35 platform and SA Pathology’s in-house VariantGrid v3 software^24^. Custom filters were implemented in both software tools to prioritise variants for review. The genome analyst was not aware of the prior diagnostic result for each case. In total, 231 and 230 SNV/indel variants were detected by Emedgene and VariantGrid v3, respectively, across the 46 validation samples, equating to approximately five variants per case requiring review. In addition, low quality CNVs that were considered artefacts after review made up a large fraction of identified variants. Cases were manually reviewed for the presence of two (likely) pathogenic variants for autosomal recessive conditions, or a single (likely) pathogenic variant for dominant conditions, either of which resulted in a ‘high chance’ classification. A total of 30 high chance cases were identified through manual review (Table 2, Supplementary Table 4). Cases with VUS, single variants in a recessive gene (carrier status), or with no variants were classified as ‘low chance’. In total, 16 low chance cases were identified. A previously reported pathogenic variant in the *ETHE1* gene was classified as a VUS. The previous diagnostic workup for this sample included biochemical analyses which were not available to the genome analyst of the NewbornsInSA study, thus resulting in a VUS classification and a low chance report (Table 2).

**Table 2:** Validation results in retrospective study

Expected Result	Details	Expected	gNBS manual review	Semi-automated gNBS	
				Auto-classified ‘For review’	Auto-classified ‘Low chance’
**High chance (n** = **31)** True positives (TP)	Cases with a NewbornsInSA reportable condition	29	28	29^a^	0
	SMA cases with *SMN1* deletion	2	2	2	0
	Variant erroneously classified as VUS	0	1^a^	0	0
**Low chance (n** = **15)** True negatives (TN)	Cases with variant(s) in a ‘red’ gene	2	2	0	2
	Cases with a single variant in a recessive gene	3	3	0	3
	Cases with non-reportable variant class (VUS)	5	5	5	0
	Known negative cases	5	5	1	4

Expected results were based on prior molecular testing. ^a^NISA_VAL_34 flagged ‘for review’ by the post-processing automation script but ETHE1 variant classified VUS in absence of biochemical testing results.

Overall, this analysis resulted in a sensitivity of 97% and a specificity of 100% (Table 3). In Emedgene, the results using the custom-developed preset group (Supplementary Table 5), and the XAI Most Likely Candidate group were mostly comparable; however, one relatively common but pathogenic variant was missed by the AI. On the other hand, the single XAI Most Likely Candidate group prioritised a total of 192 variants across all 46 cases, thus reducing the total curation effort compared to the custom preset groups developed and validated for genomic newborn screening here. Analysis using VariantGrid v3 successfully identified all previously known pathogenic SNV/indel variants but was not able to identify the two *SMN1* deletions as deletions are not yet supported in the software.

**Table 3:** Performance of the NewbornsInSA gNBS workflow

	Disease		
Test	Known Positive	Known Negative	Total
NewbornsInSA Screen Positive	30	0	30
NewbornsInSA Screen Negative	1	15	16
**Total**	**31**	**15**	**46**
**Statistic**	**Value**		
Sensitivity	97%		
Specificity	100%		
Positive Predictive Value	100% (95% CI: 88–100%)		
Negative Predictive Value	94% (95% CI: 69–99%)		
Accuracy	98%		

A known pathogenic homozygous variant NM_000063.6(*C2*):c.841_849del+19del (ClinVar: 50634) was missed by both the XAI Most Likely Candidate group and the homozygous variant prioritization strategies in sample NISA_VAL_7, due to a high population frequency of 1.135% in gnomAD v4.1.0. The variant was successfully identified in the ClinVar known pathogenic presets, where a maximum population frequency of 5% was applied to capture well-known disease variants that may occur at >1% population frequency, such as this variant and the NM_000492.3(*CFTR*):c.1521_1523del; p.(Phe508del) variant (ClinVar: 7105).

In sample NISA_VAL_34, a homozygous VUS, NM_014297.5(*ETHE1*):c.233 C > T; p.(Thr78Ile) (ClinVar: 3255123), was identified. As VUS are not reported in this screening study, this case was classified as low chance. This finding was not concordant with the prior known diagnosis of ethylmalonic encephalopathy. The diagnostic variant classification had access to biochemical testing results which upgraded the variant classification to likely pathogenic, but these results were not available to the genome analyst in this validation study and would not be routinely available in genomic newborn screening.

The two *SMN1* deletions were only detected in the DRAGEN targeted caller VCF but not in the standard CNV/SV analysis files, and could be easily missed if targeted caller files were not uploaded to the Emedgene software.

Finally, a single pathogenic *BRCA2* variant was identified in sample NISA_VAL_6. This is not a reportable finding in our study, as only bi-allelic *BRCA2* variants, associated with Fanconi anaemia (MIM# 605724), are included in the condition list for reporting. Heterozygous pathogenic *BRCA2* variants, associated with Breast cancer susceptibility (MIM# 612555), are not part of the reporting policy for this study. Both the VariantGrid and Emedgene custom preset prioritization strategies successfully filtered out this single *BRCA2* variant. The Emedgene XAI Most Likely Candidate group approach, however, flagged this single *BRCA2* variant for review as inheritance mode cannot be specified for inclusion or exclusion in the XAI Most Likely Candidate group list.

### Automating the workflow

**Fig. 1:**
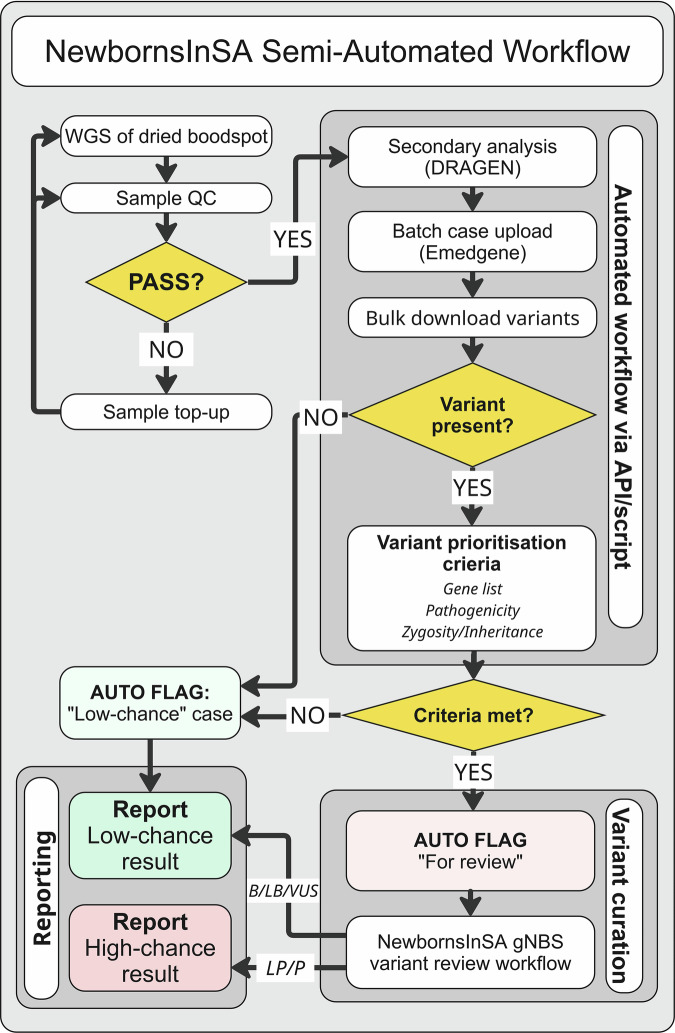
Workflow automation using a post-processing automation script via API. This workflow streamlines the review of cases by prioritizing cases with a potential high chance finding. Following tertiary analysis in Emedgene, a custom bioinformatics script interacts with the annotated variants via the API. Cases are either auto-classified as ‘Low chance’ or ‘For review’, depending on whether any variant(s) pass the prioritisation criteria (Supplementary Table 6). Variant curation for potential candidate variants is performed manually. Likely pathogenic (LP) or pathogenic (P) variants are reported as High chance results. Other variants are not reported and a Low chance result is issued. *Created in Miro*. www.miro.com/app.

To enable scaling of analysis and reduce manual curation time, a bioinformatics workflow was designed to auto-classify cases into either ‘low chance’ or ‘for review’, based on the presence or absence of candidate variants. A filtering logic was implemented through custom scripts and the Emedgene API and applied to annotated genome data (Fig. 1, Supplementary Table 6). When the bioinformatics script was applied to the 46 retrospective cases, 9 of the 15 negative cases were correctly auto-classified as ‘low chance’, requiring no further review by the genome analyst. The remaining 37 cases were flagged ‘for review’, including all 31 known positive cases (Table 2).

### Applying the NewbornsInSA gNBS workflow to prospective cases

At the conclusion of the gNBS validation study, enrolment was opened to prospective research participants in June 2024. Of the 100 samples processed and reported to date, average sequence coverage was 40x, slightly higher compared to the retrospective samples (Supplementary Table 3), with 18 DBS samples that failed to achieve 30x coverage and required top-up sequencing. Average variant numbers per case remained similar to those detected in the retrospective validation study. Manual review and automation scripts were run in parallel during this initial phase of the prospective study. The automation scripts auto-classified 53 of the total 95 negative cases or 56% as ‘low chance’, thereby immediately halving the number of cases requiring manual curation and review.

Among the 47 cases flagged ‘for review’, five high chance results have been issued (Table 4). High chance results included (likely) pathogenic variants in the *ACADM*, *GCK, KCNQ1, MT-RNR1* and *SCN5A* genes. Reports were issued to families, and for high chance results, referral to the respective clinical teams for follow-up care was made by the study team. The infant with the pathogenic *KCNQ1* variant was assessed by a tertiary paediatric cardiology service, and an ECG confirmed the presence of a prolonged QT interval. Ongoing cardiology management of the family is occurring. Similarly, the family with the *SCN5A* variant is being monitored by the paediatric cardiology team to reduce the risk of a future adverse cardiac event. The family has returned positive feedback, stating they now feel empowered to protect their baby from potential life-threatening complications associated with this condition. The *MT-RNR1* report resulted in alerts being placed in the patient’s electronic medical record to avoid future adverse aminoglycoside effects associated with this condition. By the time the *ACADM* variant was reported, the family was already managed by the metabolic unit, with the NewbornsInSA genomic report serving as an additional confirmation of an already established diagnosis. Finally, the *GCK* variant was reported to prevent unnecessary investigations once hyperglycaemia was identified in the child and to assist in managing future pregnancies if the variant was confirmed to be maternally inherited. Family studies are underway for the *KCNQ1* and *MT-RNR1* variants to determine presence in other family members.

**Table 4:** Early reporting decisions and interpretation complexities in the prospective study

Complexity	Gene/variant	Condition(s) in NISA panel	Inheritance	Assessment	Reporting decision
**None**	*ACADM:* NM_000016.6:c.985 A > G; p.(Lys329Glu) Homozygous ClinVar: 3586	Medium chain acyl CoA dehydrogenase deficiency, MIM#201450	AR	Newborn tested positive on stNBS. Family was already aware of result and being managed by the metabolic team. NewbornsInSA merely confirmed an already established diagnosis.	High chance
**Variants associated with mild phenotypes**	*GCK*: NM_000162.5:c.449 T > C; p.(Phe150Ser) Heterozygous ClinVar: 36218	MODY, type II (MIM#125851); Diabetes mellitus, noninsulin-dependent, late onset (MIM#125853); Diabetes mellitus, permanent neonatal 1 (MIM#606176); Hyperinsulinemic hypoglycemia, familial, 3 (MIM#602485)	AD/AR	Consulted local endocrinologist who found this finding relevant for the baby to avoid unnecessary management for DM and for the mother to avoid treatment for gestational diabetes that may have potential adverse outcomes during pregnancy, thus, reported as ‘high chance’.	High chance
**Typically later onset but can be relevant in baby**	*SCN5A*: NM_000335.5:c.5123 C > T; p.(Thr1708Met)Heterozygous ClinVar: 67957	Long QT syndrome 3 (MIM#603830); Brugada syndrome 1 (MIM# 601144)	AD	Strong association with Brugada Syndrome 1 in patients reported in literature; onset of disease for variant reported as 14 to 60 years of age. Consulted study’s clinical geneticist and reported due to clinical implications for both baby and family.	High chance
	*KCNQ1:* NM_000218.3:c.1552 C > T; p.(Arg518Ter) Heterozygous ClinVar: 3131	Long QT syndrome 1 (MIM# 192500)	AD	Swedish/Norwegian founder mutation that is commonly associated with a milder Long QT Syndrome 1 phenotype in symptomatic heterozygous individuals. Consulted study’s clinical geneticist and reported due to clinical implications for both baby and family.	High chance
**None**	*MT-RNR1:* NC_012920.1:m.1555 G > A Homoplasmic ClinVar: 9628	Aminoglycoside-induced deafness	MT	*MT-RNR1:*m.1555 G > A is a pharmacogenomic target with increased risk of aminoglycoside-induced deafness. Medical alert placed on the EMR to avoid aminoglycoside administration.	High chance
**Genes with both AD/AR presentation**	*PTPRQ*:c.797del; p.(Ile266Thrfs*4) Heterozygous Absent in ClinVar	Deafness, autosomal recessive 84 (MIM# 613391); Deafness, autosomal dominant 73 (MIM# 617663)	AD, AR	AD condition only reported for a single variant, c.6881 G > A p.(Trp2294*) in literature. Present variant not described in an AD setting, thus considered carrier of an AR condition.	Low chance
	*BRCA2*: NM_000059.4:c.4154 C > A; p.(Ser1385Ter) Heterozygous ClinVar: 254531	Fanconi anaemia, complementation group D1 (MIM# 605724)	AR (AD not included)	*BRCA2* is associated with multiple conditions including the AD condition breast cancer susceptibility. However, only Fanconi anaemia is included in the NewbornsInSA panel. Since proband is carrier of an AR condition only, low chance report issued to family with heterozygous *BRCA2* variant reported as ‘non-target’ finding as it is linked to susceptibility to breast cancer.	Low chance *BRCA2* variant reported as ‘non-target’ finding.
**Genes with multiple conditions where only some are relevant in gNBS**	*MITF:* NM_001354604.2:c.1273 G > A; p.(Glu425Lys) Heterozygous ClinVar: 29792	Waardenburg syndrome, type 2 A (MIM# 193510)	AD, AR	Also known as E318K, this variant is significantly associated with malignant melanoma and/or renal cell carcinoma but not Waardenburg syndrome which is the target condition for this gene.	Low chance
	*NR5A1:* NM_004959.5:c.1063 G > A; p.(Val355Met) Heterozygous ClinVar: 981141	Adrenocortical insufficiency (MIM#612964)	AD	*NR5A1* is associated with multiple, late-onset conditions, including primary ovarian insufficiency. Reported in some unrelated patients with primary ovarian insufficiency and azoospermia, as well as unaffected carriers in the literature. This does not fit target condition in NewbornsInSA panel, thus not reported.	Low chance
	*RET*: NM_020975.6:c.860 G > T; p.(Arg287Leu) Heterozygous ClinVar: 692071	Multiple endocrine neoplasia IIA (MIM# 171400); Multiple endocrine neoplasia IIB (MIM# 162300)	AD	In ClinVar (Variation ID: 692071) only reported for confirmed Hirschsprung Disease in a male neonate. This does not fit condition and disease mechanism in NewbornsInSA panel, thus not reported.	Low chance
**Complex alleles in** ***cis*** **configuration**	*BTD*: NM_001370658.1:c.1270 G > C; p.(Asp424His) Heterozygous ClinVar: 1900 *BTD*: NM_001370658.1:c.451 G > A; p.(Ala151Thr) Heterozygous ClinVar: 38298	Biotinidase deficiency (MIM#253260)	AR	Also referred to as D444H and A171T, respectively. D444H and A171T commonly occur in *cis* to form a complex allele. Proband assumed to be carrier of an AR condition only. Parental samples not available (also not feasible in population screening) at the time of DBS collection to confirm phasing.	Low chance
**Female carriers of** ***G6PD*** **deficiency**	*G6PD:* NM_001360016.2:c.949 G > A; p.(Glu317Lys) Heterozygous Female ClinVar: 10401	Glucose-6-phosphate dehydrogenase deficiency (G6PD deficiency) (MIM#300908)	XLR	WHO classified Class B variant, also known as G6PD Kerala-Kalyan variant. Found in a heterozygous, female proband (carrier only). Female carriers of G6PD deficiency are not reported in the NewbornsInSA study.	Low chance

ClinVar: ClinVar Variation ID

**Fig. 2:**
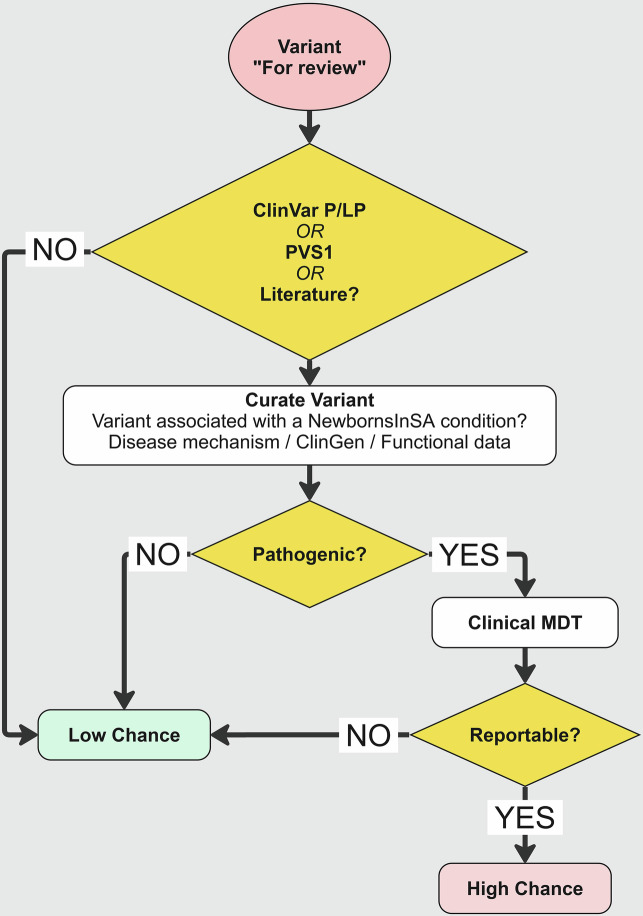
NewbornsInSA gNBS variant review workflow. Variants flagged “For review” by the post-processing automation script are manually curated using evidence in genomic databases and literature to assess variant pathogenicity. Variants are curated only against conditions specified in the NewbornsInSA gene-condition list. Factors such as disease mechanism, penetrance, prior variant classifications and functional studies and strength of gene-disease association are considered during curation. Candidate likely pathogenic/ pathogenic variants are discussed at a clinical MDT prior to reporting. *Created in Miro*. www.miro.com/app.

Many other variants were reviewed but deemed not reportable in the gNBS context, with some prompting extensive discussion among the study team (Table 4, Fig. 2). Variant interpretation in a predictive setting proved complex for a variety of reasons, including genes with both autosomal dominant and recessive modes of inheritance, genes associated with multiple conditions, complex alleles typically presenting in *cis*, genes or variants associated with mild phenotypes, and those associated with later disease onset (Table 4). For each case presented here, careful review of the literature was required to gather evidence regarding the likely condition associated with the variant, age of disease onset and severity of the identified variant. Local experts from relevant clinical subspecialties were commonly consulted as part of the MDT to assist in the final result determination. Depending on the complexity of the case, variant classification could take minutes to several hours.

## Discussion

We have successfully established and validated a genomic newborn screening assay using retrospective DBS samples and have reported on early findings from applying this workflow to a prospectively recruited infant cohort.

An important consideration for any genomic newborn screening study is which genes and variants to include for reporting. We reviewed a pre-existing gNBS gene panel, for adaptation^14^ and assessed 25 additional genes proposed by local clinical teams against our study’s inclusion criteria, with respect to clinical validity, analytical validity, and local availability of treatments and follow-up care. After detailed review, eight additional genes were included for reporting, resulting in a total of 613 genes in the final gene panel implemented here.

There is currently little international consensus on which genes should be included in gNBS studies^14^. Many gNBS studies have developed gene panels using general guiding principles based on the original Wilson and Jungner screening principles^25^. The inclusion of later onset conditions ( > 5 years of age) remains contentious, with no clear consensus on age cut-offs or criteria, leading to significant variation in gene panels across gNBS studies^14^. Members of GenSCAN^13^ and the International Consortium for Newborn Sequencing (ICoNS) are collaborating to address these important challenges.

From a technical standpoint, a significant proportion of prospective DBS samples (18%) did not meet target quality metrics and required ‘top-up’ sequencing. We are now optimising sample batching to improve this rate while also balancing sequencing cost. Our study target turn-around time for reporting is 6 to 8 weeks (42 to 56 days) which was only achieved in 41% of the first 100 cases reported. Factors such as postnatal consent (up to 2 weeks after birth), sample batching and top-up sequencing, and MDT discussions for complex variants contributed to these time delays. On average, results were returned to families on day 61 of life ( ± SD 15.9 days). Since the first 100, the laboratory now runs WGS sequencing multiple times each week, consent is primarily completed antenatally, and reporting of complex variants requires less discussion as our expertise in the area grows. These are all significant in reducing the TAT for future cases.

In this validation, all but one retrospective sample were correctly classified as high-chance or low-chance. One positive case was misclassified as low chance due to a lack of evidence available to the genome analyst to upgrade the classification from VUS to likely pathogenic. This highlights a key challenge in gNBS where follow-up functional studies are not routinely available to assist in the classification of VUS. The NewbornsInSA study is exploring the use of metabolomics as a complementary tool to gNBS and, for some variants, metabolomic data may be able to assist in variant interpretation^5^. The overall sensitivity and specificity as assessed in the retrospective validation study was 97% and 100%, respectively. However, due to small sample numbers, these results may not extrapolate to larger prospective cohorts. Importantly, no false positive screening calls were made after manual review.

Current efforts are now focused on automation to further reduce analysis time and support scalability of the gNBS approach. A post-processing automation script was implemented via the API to flag any loss-of-function and previously classified pathogenic or likely pathogenic variants. Cases with no variants for review are auto-classified as ‘low chance’. This strategy reduced the manual review burden by 56% in negative cases. With time, the criteria used in the semi-automation script can be tightened, thereby further reducing the number of variants flagged for manual review. Nevertheless, manual review remains essential, especially for variants with ambiguous interpretation, such as those in genes with multiple associated conditions or multiple modes of inheritance (AD/AR) and disease mechanisms, which often required detailed literature review and MDT discussion.

The workflow has been assessed against ISO:15189 and has been clinically accredited by the National Association of Testing Authorities (NATA). The NewbornsInSA study has now applied this validated approach to a prospectively recruited cohort, with 100 reports issued to date, including 5 high chance reports. Based on similar studies, we hypothesise a diagnostic yield of approximately 2-3% of babies^26,27^, although this rate will depend on exactly which genes and conditions are included for reporting. This yield is significantly higher than that of current Australian newborn screening programs, which have a pick-up rate of approximately 1:800 or 0.125%.

Despite rigorous validation, reporting challenges remained and were typically associated with one of the following complexities: genes with both AD and AR presentations; genes with multiple associated phenotypes, where only some conditions are relevant in the gNBS context; X-linked conditions, such as G6PD deficiency; complex alleles; secondary genes with late onset; genes/variants with mild phenotypes and multiple disease mechanisms. Our gene list includes 30 genes associated with both AD and AR conditions. For example, *TSHR* and *ABCC8* have distinct modes of action such that gain- or loss-of-function variants are associated with different dominant and recessive conditions, respectively. Other genes, such as *PTPRQ*, do not yet have an established mode of action. In either scenario, careful manual review was required, as the interpretation of candidate variants in these genes can differ significantly depending on the specific variant type. Another interpretive challenge was associated with female carriers of X-linked conditions, who can show intermediate phenotypes^28,29^ or where reporting female carriers can provide an opportunity for testing of relatives^30^. For other genes, heterozygous variants commonly occurring in *cis* configuration can lead to false positive results^26^. Since parental samples are not collected at the time of DBS collection, phasing of variants can generally not be confirmed^26^. Although some dominant genes, such as *SCN5A*, may be more commonly associated with later-onset conditions like Brugada Syndrome, reporting pathogenic variants may reduce the risk of a serious cardiovascular event which can occur in childhood through ongoing clinical management. Lastly, reduced penetrance and variable expressivity are widespread phenomena in dominant conditions^31^. As such, interpreting findings in autosomal dominant genes within a predictive setting should be done cautiously. Furthermore, identification of a dominant condition in a newborn typically leads to clinical review and consideration of predictive testing of other family members. In the NewbornsInSA study, we have set up a clinical MDT to review complex cases and reach a decision for reporting or not of variants.

Disclosing results for a condition that is not actionable until the newborn reaches a certain age can potentially cause harm, especially if there is a risk of becoming lost to follow-up^14^. The inadvertent finding of a single, pathogenic *BRCA2* mutation for an actionable, adult-onset only condition, presented an ethical dilemma. Disclosing results that do not pose a risk during childhood but are actionable only in adulthood potentially undermines the child’s autonomy^31,32^. On the other hand, these results could be clinically relevant to the parents, especially if the family were previously unaware of any genetic risk they are predisposed to^32^. In our study, the parents received the information well and sought further testing to determine their own cancer risk. This variant was detected before the XAI preset had been limited and in the clinical MDT the decision was made to report. Nevertheless, going forward, it may not always be possible to fully exclude the possibility of findings in non-target conditions and appropriate consent is important.

Safe application of gNBS requires extensive validation, ongoing monitoring, and refinement as the clinical and analytical validity of this new screening tool are established. Many studies focus on the detection of childhood-onset, treatable conditions and we have reviewed several genes proposed by local clinical teams against these criteria. We have sought strategies to minimise detection of other health information, including variants associated with later onset conditions, non-treatable conditions outside the gNBS condition list and carrier status. Such information can be made available following separate clinical requests and diagnostic re-analysis beyond the initial gNBS. The complexity of reporting genomic variants in a predictive setting, especially in the newborn period, necessitates careful review and while automation is a key enabler of population-scale screening, we settled on a semi-automated approach whereby cases with no candidate variants were auto-classified as low chance, allowing the analyst to focus attention on cases with variants flagged for review. Manual review of candidate variants for reporting was still considered necessary to ensure the quality of curation and interpretation, especially in light of some of the reporting complexities discussed here. The reporting framework developed here includes the reporting of novel variants predicted to be deleterious, so long as there is a high likelihood of the variant causing disease. An alternative strategy may limit reporting to only well-established and characterised variants, but this would likely prevent diagnosis of many rare disease patients who carry private and thus novel variants. Population- and ancestry-specific allele frequencies also have to be carefully considered. As more diverse annotation sources are generated this will help improve the quality of variant curation in a predictive screening context.

Longitudinal follow-up, including clinical utility of early detection, positive predictive value (PPV) and parental perspectives on benefits and harms are important considerations to help inform these types of programs. In this research program, we will record the outcomes of confirmatory testing, the clinical utility and change in management is measured using the C-guide tool^33^ and we capture parental perspectives through surveys at enrolment and result disclosure. The research ethics has been set up to allow re-contact of families for future research and the gNBS data are available beyond the newborn period for diagnostic re-analysis should a baby present with symptoms later in childhood.

## Methods

The retrospective validation study and prospective pilot study have been ethically approved by the Women’s and Children’s Health Network’s Human Research Ethics Committee (2022/HRE00258 and 2023/HRE00236, respectively) in accordance with the National Statement 2018 and the Declaration of Helsinki.

### Gene selection and review

Inclusion of genes in the *NewbornsInSA* study considered the guiding principles developed by The Generation study: (1) early onset disease (2) the disease-causes significant morbidity or mortality, (3) availability of effective treatment or interventions, (4) a clinically valid gene-disease association, and (5) reliable detection of disease variants using WGS^14^. The gene-condition list was based on the BabyScreen+ gene panel, available on PanelApp Australia (https://panelapp-aus.org/panels/3931/), and which includes 605 green genes, inclusive of South Australia’s stNBS conditions^15^. This panel was reviewed against local health services needs and capabilities with respect to removal or addition of conditions for screening.

### Selection of retrospective dried blood spots (DBS) for validation

A total of 46 retrospective DBS samples were chosen for this validation study to span a range of condition types, mode of inheritance (autosomal dominant, autosomal recessive, and X-linked). Cases were selected and de-identified by the study program manager.

DBS cards were included if they were < 5 years old and where the child had a previously identified molecular diagnosis. Genes present on the NewbornsInSA gene list were prioritised. To test the pipeline’s ability to filter out non-target findings, we included two positive diagnoses for a condition in a ‘red’ gene which are not screened for in NewbornsInSA. Technically challenging genes, such as *SMN1*, associated with Spinal Muscular Atrophy, a gene notorious for its poor mappability and low coverage regions, were also included in this validation. Finally, 13 ‘negative cases’ were included, of which three cases had only a single pathogenic variant in an autosomal recessive condition, five cases had only VUS and five cases had no variants in our target genes.

### DNA extraction, library preparation and whole genome sequencing

DNA was isolated from retrospective DBS samples using 3-4 punches of 3 mm diameter using Qiagen Investigator Lyse&Spin Basket Kit (catalog# 19597) and purified on the QIAsymphony automated platform (software version 5.0.4) using the QIAsymphony DSP DNA Midi Kit (catalog# 937255). DNA was quantified using Invitrogen Quant-iT PicoGreen dsDNA Assay Kit (catalog# P7589) and normalised to 1–3 ng/ul concentration on the Hamilton Microlab STARlet platform. All samples achieved sufficient DNA quality and quantity to proceed to WGS. WGS libraries were prepared and purified using the Illumina DNA PCR-Free Prep, Tagmentation Kit (catalog# 20041795) and Illumina DNA/RNA UD Indexes, Tagmentation Sets A-D (catalog# 20091654, 20091656, 20091658, and 2091660) (Low Input Method, input DNA of 25–100 ng). Normalised libraries were pooled and sequenced on the Illumina NovaSeq 6000 System (NovaSeq Control Software version 1.8.1) using NovaSeq 6000 S4 Reagent Kit v1.5 (300 cycles) (catalog# 20028312) with 2 ×150 base pair reads.

### Sequence QC and bioinformatics analysis

Bioinformatic analyses were performed using the DRAGEN v4.2 software. Raw reads were mapped to the Illumina hg38 graph genome (version 9-r3.0-1). Variant calling included SNV/indel analysis, mitochondrial variant, CNV, SV, and STR calling.

The NewbornsInSA gene list includes 6 genes known to be ‘technically challenging’: *GBA1, CYP21A2, CORO1A, SMN1, IGHM* and *F8*. *GBA1, CYP21A2, CORO1A* and *SMN1* share extensive homology with pseudogenes *GBAP1, CYP21A1P*, and the paralogous genes *CORO1B* and *SMN2* that makes short-read mapping technically challenging as reads derived from these regions do not uniquely map to the reference genome^11,12^. DRAGEN v4.2 includes targeted variant callers for *CYP21A2, GBA1* and *SMN1/SMN2*, but not yet for *CORO1A*. The *F8* gene is technically challenging due to common inversions, intron 22 inversion (Inv22) and intron 1 inversion (Inv1), which are difficult to call and annotate using the current bioinformatics pipeline. The *IGHM* gene is technically challenging as it has no RefSeq annotation in IGV which can confuse variant annotation.

To pass quality control (QC), a total usable sequencing yield of 100 Gb, equating to approximately 30x mean target coverage, was required (Supplementary Tables 2 and 3). Standard QC metrics also included the fraction of unmapped reads, PRC duplicates, and sex check. Gene-specific coverage information is available in sample-specific QC files. Samples failing coverage QC received top-up sequencing to improve coverage.

### Whole genome analysis and reporting

Variant annotation and prioritisation were performed using a dual strategy, the Illumina Emedgene v35 platform and SA Pathology’s in-house VariantGrid v3 software^24^. In Emedgene, we designed a custom set of filters with presets for autosomal recessive, autosomal dominant, de novo and x-linked modes of inheritance and uploaded the NewbornsInSA v1.110 gene list to restrict variants to within our selected genes. Variants were filtered based on variant quality (High and Moderate), frequency in population databases (AF < 1% for recessive conditions and AF < 0.1% for dominant conditions), variant effect (High or Moderate effect as defined by Ensembl’s VEP) as well as previously reported known pathogenic variants in genomic databases. Variants were classified following the American College of Medical Genetics and Genomics (ACMG) criteria. Only Pathogenic and Likely Pathogenic variants are considered for reporting in this study.

Emedgene provides a list of *Most likely candidate* variants, generated by an AI (Artificial Intelligence) analysis method. Putative pathogenic variants with established disease-gene associations are ranked by Emedgene’s automated, explainable-AI (XAI) algorithm. This XAI algorithm ranks variants from WGS data based on their molecular consequence, mode of inheritance, and allele count in phenotypically normal populations (e.g., gnomAD), followed by the known disease mechanisms (autosomal recessive, dominant or sex-linked), and known gene-disease relationships published in literature or databases.

To cross-check SNV/Indel variant calls prioritised by Emedgene, we created a NewbornsInSA analysis template in SA Pathology’s standard diagnostic variant annotation and prioritization tool, VariantGrid v3. Similar to the Emedgene custom preset group, we applied the recessive, dominant, and X-linked gene lists to the recessive, dominant, and X-linked inheritance filters using the same filtering criteria for population frequency and variant effect. Analysis in both Emedgene and VariantGrid v3 was performed in a blinded fashion, with the genome analyst not aware of the status of the case (positive or negative) nor prior molecular diagnoses.

Technical artefacts were excluded based on quality thresholds determined by the DRAGEN software for different variant types. Variants with a ‘LOW’ quality annotation or present in >10% of internally sequenced samples were excluded from further analysis, unless previously classified as Pathogenic/Likely Pathogenic in ClinVar. CNV/SV variants were limited to those 50 bp to 10 Mb in size, while SNVs/indels were limited to those with a greater than 20% variant allele frequency. Full details of the variant prioritization and filtering strategies are available in Supplementary Table 5. In April 2025, this workflow received clinical accreditation under ISO15189.

### Semi-automation of variant review

To assist with automation, a variant and case prioritization logic was implemented through custom scripts executed on annotated VCF files using the Emedgene API (Fig. 1, Supplementary Table 6). Briefly, only variants that met one or more of the following criteria were flagged for manual review: at least one ClinVar Pathogenic or Likely Pathogenic classification, predicted High impact or loss-of-function variants, missense variants of HIGH/MODERATE impact with significant splice score or in silico prediction (see also Supplementary Table 6).

## Supplementary information

Supplementary_Materials

## Data Availability

The genome sequencing data is held at SA Pathology (SA Health) as part of clinically accredited workflows and cannot be made publicly available because of patient and data protection constraints. Variants reported to families in this study are submitted to ClinVar and are thus publicly available.
