# Supplementary material for: Semi-automated genomic newborn screening highlights complexities in reporting
Source: NPJ Genom Med. 2026 Feb 10;11:13. doi: 10.1038/s41525-026-00553-4 (PMC12960665; doi:10.1038/s41525-026-00553-4)
Supplement: Supplementary file 1 — Supplementary_Materials [file 41525_2026_553_MOESM1_ESM.pdf]

Supplementary Table 1. Additional genes proposed for inclusion but not meeting criteria for gNBS. Clinical validity is based on ClinGen classifications of the strength of evidence for a gene-disease relationship.

Gene	Condition	Reason(s) for not including
ABCA4	Cone-rod dystrophy 3 (MIM#604116) (AR)	No specific treatment(s) available; Variable age of onset
ACE	Renal tubular dysgenesis (MIM#267430) (AR)	No specific treatment(s) available
AKAP9	Long QT Syndrome 11 (MIM#611820) (AD)	Variable (usually late) age of onset; Absent or limited clinical validity (ClinGen)
CACNA1C	Long QT syndrome 8 (MIM#618447) (AD)<br>Timothy Syndrome (MIM#601005) (AD)	Variable (usually late) age of onset; Absent or limited clinical validity (ClinGen)
CACNA1F	Cone-rod dystrophy 3 (MIM#300476) (XLR)	No specific treatment(s) available
CRELD1	Cardiac atrioventricular septal defect (MIM#606217) (AD)	Limited clinical validity (ClinGen)
DMD*	Duchenne muscular dystrophy* (MIM# 310200) (XLR)	No specific treatment(s) available
ELN	Williams Syndrome (MIM#194050) (AD)	No clinical validity (ClinGen)
KCNE1	Long QT Syndrome 5 (MIM#613695) (AD)	Limited clinical validity (ClinGen)
KCNE2	Long QT Syndrome 6 (MIM#613693) (AD)	Limited clinical validity (ClinGen)
KCNJ5	Long QT Syndrome 13 (MIM#613485) (AD)	Variable (usually late) age of onset; Absent or limited clinical validity (ClinGen)
MLXIPL	Williams Syndrome (MIM#194050) (AD)	No clinical validity (ClinGen)
PKD1	Polycystic kidney disease 1 (MIM#173900) (AD)	Late age of onset
SCN4B	Long QT Syndrome 10 (MIM#611819) (AD)	Variable (usually late) age of onset; Absent or limited clinical validity (ClinGen)
SNTA1	Long QT syndrome 12 (MIM#612955) (AD)	Variable (usually late) age of onset; Absent or limited clinical validity (ClinGen)
TNNT1	Nemaline myopathy 5C (MIM#620389) (AD)	Variable (usually late) age of onset; Absent or limited clinical validity (ClinGen)
TNNT3	Arthrogryposis, distal, type 2B2 (MIM#618435) (AD)	Variable (usually late) age of onset; Absent or limited clinical validity (ClinGen)

**No clinical trial available in South Australia*

Supplementary Table 2. WGS data output and base call quality metrics of 46 retrospective samples.

Sample	Coverage (x)	Duplicate Fraction	Unmapped Fraction	Yield (gigabase)	% Q30
NISA_VAL_1	28	0.26	0.0024	113.49	90.23
NISA_VAL_2	29	0.26	0.0024	115.24	90.24
NISA_VAL_3	29	0.27	0.0023	123.89	90.44
NISA_VAL_4	30	0.27	0.0026	130.23	89.79
NISA_VAL_5	32	0.28	0.0022	140.11	90.50
NISA_VAL_6	40	0.28	0.0021	171.89	90.73
NISA_VAL_7	32	0.27	0.0020	136.16	90.80
NISA_VAL_8	27	0.26	0.0022	114.90	90.62
NISA_VAL_9	26	0.26	0.0019	108.64	90.55
NISA_VAL_10	22	0.28	0.0017	95.95	90.73
NISA_VAL_11	34	0.28	0.0023	147.15	90.40
NISA_VAL_12	30	0.29	0.0024	130.90	90.15
NISA_VAL_13	27	0.27	0.0022	115.40	90.47
NISA_VAL_14	31	0.27	0.0026	132.64	90.04
NISA_VAL_15	37	0.27	0.0022	160.50	90.29
NISA_VAL_16	32	0.28	0.0020	138.28	90.68
NISA_VAL_17	29	0.27	0.0024	121.27	90.30
NISA_VAL_18	40	0.27	0.0022	169.23	90.48
NISA_VAL_19	28	0.26	0.0025	115.77	89.86
NISA_VAL_20	32	0.28	0.0022	138.68	90.35
NISA_VAL_21	32	0.29	0.0015	140.77	90.36
NISA_VAL_22	32	0.27	0.0020	138.34	90.81
NISA_VAL_23	34	0.27	0.0020	146.44	90.47
NISA_VAL_24	35	0.18	0.0021	130.19	91.45
NISA_VAL_25	33	0.18	0.0018	124.75	91.79
NISA_VAL_26	35	0.18	0.0019	130.71	91.77
NISA_VAL_27	38	0.18	0.0020	140.66	91.52
NISA_VAL_28	31	0.18	0.0021	117.59	91.45
NISA_VAL_29	31	0.17	0.0017	116.05	91.83
NISA_VAL_30	36	0.17	0.0019	134.47	91.55
NISA_VAL_31	35	0.17	0.0021	133.18	91.36
NISA_VAL_32	36	0.17	0.0020	135.63	91.41
NISA_VAL_33	35	0.17	0.0017	131.59	91.73
NISA_VAL_34	23	0.17	0.0020	87.26	91.24
NISA_VAL_35	41	0.18	0.0020	152.46	91.70
NISA_VAL_36	38	0.18	0.0021	145.08	91.59
NISA_VAL_37	34	0.17	0.0018	127.38	91.85
NISA_VAL_38	30	0.17	0.0020	111.76	91.38

NISA_VAL_39	35	0.17	0.0018	133.16	91.75
NISA_VAL_40	33	0.18	0.0019	122.09	91.49
NISA_VAL_41	38	0.17	0.0019	142.12	91.61
NISA_VAL_42	43	0.18	0.0021	161.74	91.67
NISA_VAL_43	34	0.18	0.0020	130.45	91.36
NISA_VAL_44	38	0.17	0.0018	138.16	91.20
NISA_VAL_45	42	0.18	0.0020	156.62	91.60
NISA_VAL_46	41	0.19	0.0020	155.27	91.72
Average	**33x**	**0.22**	**0.0021**	**132.70Gb**	**90.99**

Supplementary Table 3. WGS data output and base call quality metrics of 100 prospective samples.

Sample	Coverage (x)	Duplicate Fraction	Unmapped Fraction	Yield (gigabase)	% Q30
NISA_Prosp_1	32	0.14	0.03	114.10	90.97
NISA_Prosp_2	32	0.13	0.03	113.83	91.17
NISA_Prosp_3	30	0.14	0.03	107.46	90.78
NISA_Prosp_4	27	0.13	0.03	95.65	91.12
NISA_Prosp_5	32	0.09	0.03	107.63	91.49
NISA_Prosp_6	29	0.09	0.03	97.40	89.45
NISA_Prosp_7	32	0.09	0.03	107.89	90.66
NISA_Prosp_8	32	0.09	0.03	104.94	90.01
NISA_Prosp_9	32	0.09	0.03	106.32	89.82
NISA_Prosp_10	30	0.10	0.03	101.61	90.19
NISA_Prosp_11	40	0.08	0.02	134.93	90.81
NISA_Prosp_12	39	0.08	0.02	131.01	90.9
NISA_Prosp_13	33	0.14	0.02	118.50	92.28
NISA_Prosp_14	30	0.16	0.03	110.96	92.71
NISA_Prosp_15	37	0.16	0.03	134.20	91.79
NISA_Prosp_16	30	0.14	0.03	104.76	92.25
NISA_Prosp_17	33	0.14	0.02	118.50	92.28
NISA_Prosp_18	30	0.16	0.03	110.96	92.71
NISA_Prosp_19	28	0.15	0.03	103.07	91.74
NISA_Prosp_20	38	0.14	0.03	137.11	91.57
NISA_Prosp_21	41	0.57	0.04	306.37	89.24
NISA_Prosp_22	27	0.13	0.07	103.21	90.85
NISA_Prosp_23	33	0.08	0.03	112.02	90.02
NISA_Prosp_24	36	0.09	0.03	120.27	90.23
NISA_Prosp_25	50	0.08	0.03	167.64	90.34
NISA_Prosp_26	38	0.11	0.02	129.51	89.52
NISA_Prosp_27	44	0.09	0.03	145.80	91.65
NISA_Prosp_28	34	0.07	0.03	109.90	91.49
NISA_Prosp_29	45	0.09	0.03	148.08	91.55
NISA_Prosp_30	35	0.08	0.03	117.96	91.54
NISA_Prosp_31	32	0.07	0.03	103.92	91.41
NISA_Prosp_32	52	0.08	0.03	175.53	90.93
NISA_Prosp_33	36	0.10	0.02	122.13	90.91
NISA_Prosp_34	31	0.09	0.02	104.00	90.53
NISA_Prosp_35	36	0.10	0.02	119.45	90.66
NISA_Prosp_36	46	0.11	0.03	158.67	89.42
NISA_Prosp_37	39	0.10	0.02	131.77	90.45
NISA_Prosp_38	36	0.08	0.02	116.53	90.88

NISA_Prosp_39	35	0.07	0.02	114.45	90.09
NISA_Prosp_40	44	0.09	0.03	146.04	90.29
NISA_Prosp_41	51	0.09	0.02	173.05	90.65
NISA_Prosp_42	35	0.08	0.02	117.95	90.35
NISA_Prosp_43	48	0.09	0.02	160.12	90.31
NISA_Prosp_44	36	0.08	0.02	118.45	90.55
NISA_Prosp_45	35	0.09	0.03	118.19	90.99
NISA_Prosp_46	45	0.09	0.02	151.65	90.54
NISA_Prosp_47	35	0.07	0.02	115.49	90.42
NISA_Prosp_48	34	0.08	0.03	112.25	89.8
NISA_Prosp_49	33	0.11	0.02	111.01	91.01
NISA_Prosp_50	35	0.11	0.03	119.10	90.89
NISA_Prosp_51	34	0.11	0.03	114.62	90.84
NISA_Prosp_52	39	0.11	0.02	132.63	90.47
NISA_Prosp_53	33	0.11	0.02	112.50	90.45
NISA_Prosp_54	42	0.12	0.02	143.62	90.86
NISA_Prosp_55	36	0.12	0.03	122.73	91
NISA_Prosp_56	35	0.11	0.02	117.45	90.61
NISA_Prosp_57	31	0.10	0.02	105.62	90.51
NISA_Prosp_58	91	0.20	0.03	344.46	91.97
NISA_Prosp_59	85	0.20	0.02	323.68	91.85
NISA_Prosp_60	75	0.18	0.03	282.08	91.66
NISA_Prosp_61	83	0.19	0.02	311.71	91.75
NISA_Prosp_62	66	0.17	0.03	240.65	91.95
NISA_Prosp_63	98	0.21	0.03	375.37	92.39
NISA_Prosp_64	63	0.18	0.02	237.04	92.03
NISA_Prosp_65	57	0.18	0.02	214.13	92.15
NISA_Prosp_66	79	0.19	0.02	297.87	92.1
NISA_Prosp_67	80	0.19	0.02	301.12	92.07
NISA_Prosp_68	38	0.16	0.03	139.62	92.1
NISA_Prosp_69	41	0.17	0.03	151.09	92.08
NISA_Prosp_70	35	0.15	0.03	129.31	91.41
NISA_Prosp_71	32	0.14	0.03	116.85	91.83
NISA_Prosp_72	41	0.17	0.03	150.13	91.61
NISA_Prosp_73	36	0.17	0.03	135.13	92.1
NISA_Prosp_74	28	0.15	0.03	101.73	91.96
NISA_Prosp_75	39	0.18	0.03	145.21	92.2
NISA_Prosp_76	32	0.15	0.03	114.39	91.6
NISA_Prosp_77	36	0.14	0.03	129.83	91.89
NISA_Prosp_78	37	0.18	0.03	135.69	91.72
NISA_Prosp_79	37	0.16	0.03	132.59	92.11
NISA_Prosp_80	38	0.18	0.04	140.96	92.6

NISA_Prosp_81	46	0.19	0.03	171.82	92.24
NISA_Prosp_82	33	0.18	0.04	124.00	92.51
NISA_Prosp_83	39	0.18	0.03	144.21	92.11
NISA_Prosp_84	38	0.19	0.03	146.88	92.17
NISA_Prosp_85	35	0.19	0.03	133.04	91.94
NISA_Prosp_86	36	0.17	0.03	135.80	92.27
NISA_Prosp_87	29	0.17	0.03	108.21	92.53
NISA_Prosp_88	29	0.17	0.03	108.56	92.05
NISA_Prosp_89	34	0.19	0.03	131.33	92.33
NISA_Prosp_90	34	0.18	0.03	129.54	92.13
NISA_Prosp_91	36	0.19	0.03	134.60	92.45
NISA_Prosp_92	46	0.10	0.18	158.87	88.99
NISA_Prosp_93	39	0.15	0.19	140.52	89.40
NISA_Prosp_94	35	0.09	0.20	119.98	88.87
NISA_Prosp_95	52	0.10	0.25	179.14	89.27
NISA_Prosp_96	38	0.16	0.16	139.23	89.91
NISA_Prosp_97	33	0.16	0.14	122.70	89.91
NISA_Prosp_98	35	0.15	0.16	125.28	89.53
NISA_Prosp_99	33	0.15	0.18	120.94	89.73
NISA_Prosp_100	34	0.16	0.14	126.23	90.10
**Average**	**40x**	**0.14**	**0.04**	**145.12Gb**	**91.13%**

Supplementary Table 4. Analysis of 46 retrospective samples in Emedgene v35 and VariantGrid v3.

NISA ID	Detected variants and Diagnosis	Identified in Emedgene v35	Identified in VariantGrid v3	Concordant with previous test results
NISA_VAL_1	**NM_000171.4(GLRA1):c.896G>A (p.Arg299Gln)**<br>High chance - Hyperekplexia, hereditary 1, autosomal dominant or recessive	Yes	Yes	Yes
NISA_VAL_2	**NM_000143.4(FH):c.[1457del];[1431_1433dup]; p.[Ala486ValfsTer16];[Lys477dup]**<br>High chance - Fumurase deficiency	Yes	Yes	Yes
NISA_VAL_3	**NM_000518.5(HBB):c.20A>T (p.Glu7Val)**<br>High chance - Sickle cell anaemia	Yes	Yes	Yes
NISA_VAL_4	**SMN1 deletion**<br>High chance – Spinal muscular atrophy	Yes	No	Yes (Note: deletions not in VG3)
NISA_VAL_5	**NM_000463.3(UGT1A1):c.[1220del];[524T>A]; p.[Lys407fs];[Leu175Gln]**<br>High chance - Crigler-Najjar syndrome	Yes	Yes	Yes
NISA_VAL_6	**Low chance**	Yes	Yes	Yes
NISA_VAL_7	**NM_000063.6(C2):c.841_849+19del**<br>High chance – C2 deficiency	Yes	Yes	Yes
NISA_VAL_8	**NM_000182.5(HADHA): c.1528G>C (p.Glu510Gln)**<br>High chance - LCHAD deficiency and Mitochondrial trifunctional protein deficiency	Yes	Yes	Yes
NISA_VAL_9	**SMN1 deletion**<br>High chance – Spinal muscular atrophy	Yes	No	Yes (Note: deletions not in VG3)
NISA_VAL_10	**NM_000016.6(ACADM):c.985A>G (p.Lys329Glu)**<br>**NM_000016.6(ACADM):c.999_1011dup (p.Gln338LeufsTer3)**	Yes	Yes	Yes

	High chance – MCAD deficiency			
NISA_VAL_11	**NM_000218.3(KCNQ1): c.1022C>A (p.Ala341Glu)**<br>High chance - Long QT Syndrome 1	Yes	Yes	Yes
NISA_VAL_12	**TSHR c.1358T>C, pMet453Thr**<br>Low chance - Hypothyroidism, congenital, nongoitrous, 1 (AR); single variant in a recessive gene	Yes	Yes	Yes
NISA_VAL_13	**NM_000317.3(PTS) c.286G>A (p.Asp96Asn)**<br>**NM_000317.3(PTS): c.351C>A (p.Asn117Lys)**<br>High chance - Hyperphenylalaninemia, BH4-deficient, A	Yes	Yes	Yes
NISA_VAL_14	**NM_000065.5(C6): c.1153G>T (p.Glu385Ter)**<br>High chance – C6 deficiency	Yes	Yes	Yes
NISA_VAL_15	**AVPR2 (?_153905146)_(153906728_?)del**<br>Low chance - Diabetes insipidus, nephrogenic (proband is female; only biallelic reported in females)	Yes	Yes	Yes
NISA_VAL_16	**NM_000441.2(SLC26A4): c.2015G>A (p.Gly672Glu)**<br>**NM_000441.2(SLC26A4): c.1343C>T p.Ser448Leu**<br>High chance - Deafness, autosomal recessive 4, with enlarged vestibular aqueduct<br>Pendred syndrome	Yes	Yes	Yes
NISA_VAL_17	**Low chance**	Yes	Yes	Yes
NISA_VAL_18	**NM_000383.4(AIRE): c.349del (p.Ala117ProfsTer30)**<br>**NM_000383.4(AIRE): c.967_979del (p.Leu323SerfsTer51)**<br>High chance - Autoimmune polyendocrinopathy syndrome , type I, with or without reversible metaphyseal dysplasia,	Yes	Yes	Yes
NISA_VAL_19	**NM_000516.7(GNAS): c.845T>G (p.Leu282Arg)**<br>High chance - Pseudopseudohypoparathyroidism;	Yes	Yes	Yes

	Pseudohypoparathyroidism			
NISA_VAL_20	**NM_000155.4(GALT):c.584T>C (p.Leu195Pro)**<br>**NM_000155.4(GALT):c.563A>G (p.Gln188Arg)**<br>High chance - Galactosaemia	Yes	Yes	Yes
NISA_VAL_21	**NM_000151.4(G6PC1):c.562G>A (p.Gly188Ser)**<br>High chance - Glycogen storage disease Ia	Yes	Yes	Yes
NISA_VAL_22	**NM_000352.6(ABCC8):c.2857C>T (p.Gln953Ter)**<br>**NM_000352.6(ABCC8):c.3130_3149del (p.Thr1044fs)**<br>High chance - Hyperinsulinemic hypoglycemia, familial	Yes	Yes	Yes
NISA_VAL_23	**NM_000642.3(AGL):c.3816_3817del (p.Gly1273fs)**<br>High chance - Glycogen storage disease IIIa	Yes	Yes	Yes
NISA_VAL_24	**NM_024426.6(WT1):c.410del (p.Pro137ArgfsTer26)**<br>High chance – Wilms Tumour type 1	Yes	Yes	Yes
NISA_VAL_25	**NM_017654.4(SAMD9):c.2414A>G (p.Asp805Gly)**<br>High chance - MIRAGE syndrome	Yes	Yes	Yes
NISA_VAL_26	**NM_000284.4(PDHA1):c.905G>A (p.Arg302His)**<br>High chance - Pyruvate dehydrogenase E1-alpha deficiency	Yes	Yes	Yes
NISA_VAL_27	**NM_000277.3(PAH):c.1045T>C (p.Ser349Pro)**<br>**NM_000277.3(PAH):c.117C>G (p.Phe39Leu)**<br>High chance - Phenylketonuria	Yes	Yes	Yes
NISA_VAL_28	**NM_000277.3(PAH):c.1315+1G>A**<br>**NM_000277.3(PAH):c.1222C>T (p.Arg408Trp)**<br>High chance - Phenylketonuria	Yes	Yes	Yes
NISA_VAL_29	**NM_000016.6(ACADM):c.799G>A (p.Gly267Arg)**<br>**NM_000016.6(ACADM):c.797A>G (p.Asp266Gly)**<br>High chance - MCADD	Yes	Yes	Yes

NISA_VAL_30	**NM_000018.4(ACADVL):c.1226C>T (p.Thr409Met)**<br>Low chance – VUS not reportable	Yes	Yes	Yes
NISA_VAL_31	**NM_001363711.2(DUOX2):c.2895_2898del (p.Phe966SerfsTer29)**<br>**NM_001363711.2(DUOX2):c.1479dup (p.Gly494TrpfsTer22)**<br>High chance - Thyroid dyshormonogenesis 6	Yes	Yes	Yes
NISA_VAL_32	**NM_000016.6(ACADM):c.1129G>A (p.Gly377Arg)**<br>**NM_000016.6(ACADM):c.600-18G>A**<br>Low chance - ACADM c.1129G>A is a VUS not reportable	Yes	Yes	Yes
NISA_VAL_33	**Low chance**	Yes	Yes	Yes
*NISA_VAL_34	**NM_014297.5(ETHE1):c.233C>T (p.Thr78Ile)**<br>High chance – VUS upgraded to Likely Pathogenic	Yes	Yes	Yes
NISA_VAL_35	**NM_000532.5(PCCB):c.821T>C (p.Phe274Ser)**<br>Low chance – VUS not reportable	Yes	Yes	Yes
NISA_VAL_36	**NM_001363711.2(DUOX2):c.127A>T (p.Asn43Tyr)**<br>**NM_001363711.2(DUOX2):c.2654G>T (p.Arg885Leu)**<br>High chance - Thyroid dyshormonogenesis 6	Yes	Yes	Yes
NISA_VAL_37	**NM_000016.6(ACADM):c.985A>G (p.Lys329Glu)**<br>**NM_000016.6(ACADM):c.558T>A (p.Asn186Lys)**<br>High chance - MCADD	Yes	Yes	Yes
NISA_VAL_38	**NM_001363711.2(DUOX2):c.1873C>T (p.Arg625Ter)**<br>**NM_001363711.2(DUOX2):c.1709A>T (p.Gln570Leu)**<br>High chance - Thyroid dyshormonogenesis 6	Yes	Yes	Yes
NISA_VAL_39	**MYH7 c.475G> p.Asp159Asn**<br>Low chance – Single variant in a recessive gene	Yes	Yes	Yes
NISA_VAL_40	**NM_000531.6(OTC):c.717G>T (p.Glu239Asp)**<br>High chance - Ornithine transcarbamylase deficiency	Yes	Yes	Yes

NISA_VAL_41	**NM_000547.6(TPO):c.239C>T (p.Pro80Leu)**<br>**NM_001206744.2(TPO):c.1184_1187dup (p.Ala397fsTer76)**<br>Low chance - c.239C>T is a VUS not reportable	Yes	Yes	Yes
NISA_VAL_42	**Low chance**	Yes	Yes	Yes
NISA_VAL_43	**NM_000388.4(CASR):c.379G>A (p.Glu127Lys)**<br>High chance - Hypocalcemia, autosomal dominant	Yes	Yes	Yes
NISA_VAL_44	**Low chance**	Yes	Yes	Yes
NISA_VAL_45	**Low chance**	Yes	Yes	Yes
NISA_VAL_46	**Low chance**	Yes	Yes	Yes
**Result concordance**	**45/46**			

*NISA_VAL_34 flagged ‘for review’ by the post-processing automation script but *ETHE1* variant classified VUS in absence of biochemical testing results.

Supplementary Table 5. NewbornsInSA presets and parameters in Emedgene.

Preset	Filtering
Most likely by AI	**Variant effect filters:**<br>Exclusion classification: clinvar, curate, Benign, Likely Benign, 'criteria provided, multiple submitters, no conflicts'<br>**Other filters:**<br>aa_tags: most_likely<br>gene_list_id: 4622
AR Homozygous	**Quality filters:**<br>Allele bias: >= 20<br>Depth: >= 10<br>50 <= Length <= 250000000<br>MQM: >= 45<br>Quality: HIGH, MODERATE<br>**Polymorphisms filters:**<br>Max all AF: <= 0.01<br>Org db AF, SA-Pathology-nisa-hg38-noise-any-cnv: <= 0.1<br>Org db AF, SA-Pathology-nisa-hg38-noise-any-manta: <= 0.1<br>Org db AF, SA-Pathology-nisa-hg38-noise-any-snv: <= 0.1<br>**Variant effect filters:**<br>Exclusion classification: clinvar, curate, Benign, Likely Benign, 'criteria provided, multiple submitters, no conflicts', 'criteria provided, single submitter'<br>Severity: HIGH, MODERATE<br>**Zygosity filters:**<br>Father Zygosity: HET, REF, no_coverage<br>Mother Zygosity: HET, REF, no_coverage<br>Proband: HOM<br>**Search Filters:**<br>Variant: chr1,1,1000000000000, chr2,1,1000000000000, chr3,1,1000000000000, chr4,1,1000000000000, chr4,1,1000000000000 ...<br>**Other filters:**<br>gene_list_id: 4621
AR Compound Heterozygous	**Quality filters:**<br>Allele bias: >= 20<br>Depth: >= 10<br>50 <= Length <= 250000000<br>MQM: >= 45<br>Quality: HIGH, LOW, MODERATE<br>**Polymorphisms filters:**<br>Max all AF: <= 0.01<br>Org db AF, SA-Pathology-nisa-hg38-noise-any-cnv: <= 0.1<br>Org db AF, SA-Pathology-nisa-hg38-noise-any-manta: <= 0.1<br>Org db AF, SA-Pathology-nisa-hg38-noise-any-snv: <= 0.1<br>**Variant effect filters:**<br>Exclusion classification: clinvar, curate, Benign, Likely Benign, 'criteria provided, multiple submitters, no conflicts', 'criteria provided, single submitter'

	Severity: HIGH, MODERATE<br>**Inheritance filters:**<br>AR Comp Het: True<br>**Other filters:**<br>gene_list_id: 4621
AD Heterozygous	**Quality filters:**<br>Allele bias: >= 20<br>Depth: >= 10<br>50 <= Length <= 250000000<br>MQM: >= 45<br>Quality: HIGH, MODERATE<br>**Polymorphisms filters:**<br>All Hom/Hemi: <= 10<br>Max all AF: <= 0.001<br>Org db AF, SA-Pathology-nisa-hg38-noise-any-cnv: <= 0.1<br>Org db AF, SA-Pathology-nisa-hg38-noise-any-manta: <= 0.1<br>Org db AF, SA-Pathology-nisa-hg38-noise-any-snv: <= 0.1<br>**Variant effect filters:**<br>Exclusion classification: clinvar, curate, Benign, Likely Benign, 'criteria provided, multiple submitters, no conflicts', 'reviewed by expert panel', 'criteria provided, single submitter'<br>Severity: HIGH, MODERATE<br>**Zygosity filters:**<br>Father Zygosity: HET, REF, no_coverage<br>Mother Zygosity: HET, REF, no_coverage<br>Proband: HET<br>**Other filters:**<br>gene_list_id: 3543
De novo	**Quality filters:**<br>Allele bias: >= 20<br>Depth: >= 10<br>50 <= Length <= 250000000<br>MQM: >= 45<br>Quality: HIGH, MODERATE<br>**Polymorphisms filters:**<br>All Hom/Hemi: <= 10<br>Max all AF: <= 0.0001<br>Org db AF, SA-Pathology-nisa-hg38-noise-any-cnv: <= 0.1<br>Org db AF, SA-Pathology-nisa-hg38-noise-any-manta: <= 0.1<br>Org db AF, SA-Pathology-nisa-hg38-noise-any-snv: <= 0.1<br>**Variant effect filters:**<br>Exclusion classification: clinvar, curate, Benign, Likely Benign, 'criteria provided, multiple submitters, no conflicts'<br>Severity: HIGH, MODERATE<br>**Zygosity filters:**<br>Father Zygosity: REF, no_coverage<br>Mother Zygosity: REF, no_coverage<br>Proband: HET, HOM<br>**Other filters:**<br>gene_list_id: 3544

XLR Male	**Quality filters:**<br>Allele bias: >= 20<br>Depth: >= 10<br>50 <= Length <= 250000000<br>MQM: >= 45<br>Quality: HIGH, MODERATE<br>**Polymorphisms filters:**<br>Max all AF: <= 0.01<br>Org db AF, SA-Pathology-nisa-hg38-noise-any-cnv: <= 0.1<br>Org db AF, SA-Pathology-nisa-hg38-noise-any-manta: <= 0.1<br>Org db AF, SA-Pathology-nisa-hg38-noise-any-snv: <= 0.1<br>**Variant effect filters:**<br>Exclusion classification: clinvar, curate, Benign, Likely Benign, 'criteria provided, multiple submitters, no conflicts', 'reviewed by expert panel', 'criteria provided, single submitter'<br>Severity: HIGH, MODERATE<br>**Zygosity filters:**<br>Father Zygosity: REF, no_coverage<br>Mother Zygosity: HET, no_coverage<br>Proband: HOM, HET<br>**Search Filters:**<br>Inheritance Mode: XLR,X-linked recessive<br>Variant: chrX,1,1000000000000<br>**Other filters:**<br>gene_list_id: 3549
XLR Female	**Quality filters:**<br>Allele bias: >= 20<br>Depth: >= 10<br>50 <= Length <= 250000000<br>MQM: >= 45<br>Quality: HIGH, MODERATE<br>**Polymorphisms filters:**<br>Max all AF: <= 0.01<br>Org db AF, SA-Pathology-nisa-hg38-noise-any-cnv: <= 0.1<br>Org db AF, SA-Pathology-nisa-hg38-noise-any-manta: <= 0.1<br>Org db AF, SA-Pathology-nisa-hg38-noise-any-snv: <= 0.1<br>**Variant effect filters:**<br>Exclusion classification: clinvar, curate, Benign, Likely Benign, 'criteria provided, multiple submitters, no conflicts', 'reviewed by expert panel', 'criteria provided, single submitter'<br>Severity: HIGH, MODERATE<br>**Zygosity filters:**<br>Father Zygosity: HOM, no_coverage<br>Mother Zygosity: HET, no_coverage<br>Proband: HOM<br>**Search Filters:**<br>Variant: chrX,1,1000000000000<br>**Other filters:**<br>gene_list_id: 3549
XLD	**Quality filters:**

	Allele bias: >= 20<br>Depth: >= 10<br>50 <= Length <= 250000000<br>MQM: >= 45<br>Quality: HIGH, MODERATE<br>**Polymorphisms filters:**<br>Max all AF: <= 0.001<br>Org db AF, SA-Pathology-nisa-hg38-noise-any-cnv: <= 0.1<br>Org db AF, SA-Pathology-nisa-hg38-noise-any-manta: <= 0.1<br>Org db AF, SA-Pathology-nisa-hg38-noise-any-snv: <= 0.1<br>**Variant effect filters:**<br>Exclusion classification: clinvar, curate, Benign, Likely Benign, 'criteria provided, multiple submitters, no conflicts', 'reviewed by expert panel', 'criteria provided, single submitter'<br>Severity: HIGH, MODERATE<br>**Zygosity filters:**<br>Father Zygosity: REF, no_coverage<br>Mother Zygosity: HET, REF, no_coverage<br>Proband: HET<br>**Search Filters:**<br>Variant: chrX,1,1000000000000<br>**Other filters:**<br>gene_list_id: 3548
ClinVar Path AR Homozygous	**Quality filters:**<br>Depth: >= 10<br>50 <= Length <= 250000000<br>MQM: >= 45<br>Quality: HIGH, LOW, MODERATE<br>**Polymorphisms filters:**<br>Max all AF: <= 0.05<br>Org db AF, SA-Pathology-nisa-hg38-noise-any-cnv: <= 0.1<br>Org db AF, SA-Pathology-nisa-hg38-noise-any-manta: <= 0.1<br>Org db AF, SA-Pathology-nisa-hg38-noise-any-snv: <= 0.1<br>**Variant effect filters:**<br>Exclusion classification: clinvar, curate, Benign, Likely Benign, 'criteria provided, multiple submitters, no conflicts'<br>Known pathogenic variants: True<br>Severity: HIGH, MODERATE<br>**Zygosity filters:**<br>Proband: HOM<br>**Other filters:**<br>gene_list_id: 4621
ClinVar Path AR Compound Heterozygous	**Quality filters:**<br>Depth: >= 10<br>50 <= Length <= 250000000<br>MQM: >= 45<br>Quality: HIGH, LOW, MODERATE<br>**Polymorphisms filters:**<br>Max all AF: <= 0.05<br>Org db AF, SA-Pathology-nisa-hg38-noise-any-cnv: <= 0.1

	Org db AF, SA-Pathology-nisa-hg38-noise-any-manta: <= 0.1<br>Org db AF, SA-Pathology-nisa-hg38-noise-any-snv: <= 0.1<br>**Variant effect filters:**<br>Exclusion classification: clinvar, curate, Benign, Likely Benign, 'criteria provided, multiple submitters, no conflicts'<br>Known pathogenic variants: True<br>Severity: HIGH, MODERATE<br>**Inheritance filters:**<br>AR Comp Het: True<br>**Other filters:**<br>gene_list_id: 4621
ClinVar Path AD	**Quality filters:**<br>Depth: >= 10<br>50 <= Length <= 250000000<br>MQM: >= 45<br>Quality: HIGH, LOW, MODERATE<br>**Polymorphisms filters:**<br>Max all AF: <= 0.05<br>Org db AF, SA-Pathology-nisa-hg38-noise-any-cnv: <= 0.1<br>Org db AF, SA-Pathology-nisa-hg38-noise-any-manta: <= 0.1<br>Org db AF, SA-Pathology-nisa-hg38-noise-any-snv: <= 0.1<br>**Variant effect filters:**<br>Exclusion classification: clinvar, curate, Benign, Likely Benign, 'criteria provided, multiple submitters, no conflicts'<br>Known pathogenic variants: True<br>Severity: HIGH, MODERATE<br>**Zygosity filters:**<br>Proband: HET<br>**Other filters:**<br>gene_list_id: 3543
ClinVar XL	**Quality filters:**<br>Depth: >= 10<br>50 <= Length <= 250000000<br>MQM: >= 45<br>Quality: HIGH, LOW, MODERATE<br>**Polymorphisms filters:**<br>Max all AF: <= 0.05<br>Org db AF, SA-Pathology-nisa-hg38-noise-any-cnv: <= 0.1<br>Org db AF, SA-Pathology-nisa-hg38-noise-any-manta: <= 0.1<br>Org db AF, SA-Pathology-nisa-hg38-noise-any-snv: <= 0.1<br>**Variant effect filters:**<br>Exclusion classification: clinvar, curate, Benign, Likely Benign, 'criteria provided, multiple submitters, no conflicts'<br>Known pathogenic variants: True<br>Severity: HIGH, MODERATE<br>**Inheritance filters:**<br>XLR: True<br>**Other filters:**<br>gene_list_id: 3549
CNV/SV AR	**Quality filters:**

	Bin: >= 1<br>Depth: >= 10<br>50 <= Length <= 10000000<br>MQM: >= 45<br>Quality: HIGH, MODERATE<br>**Polymorphisms filters:**<br>Max all AF: <= 0.03<br>Org db AF, SA-Pathology-nisa-hg38-noise-any-cnv: <= 0.1<br>Org db AF, SA-Pathology-nisa-hg38-noise-any-manta: <= 0.1<br>Org db AF, SA-Pathology-nisa-hg38-noise-any-snv: <= 0.1<br>**Variant type filters:**<br>Variant type: del, dup, ins<br>Variant effect filters:<br>Severity: HIGH, MODERATE<br>**Gene filters:**<br>Candidate genes: True<br>**Zygosity filters:**<br>Proband: HET, HOM<br>**Search Filters:**<br>Gene List: NewbornsInSA_AR_ADAR_v1.1
CNV/SV AD	**Quality filters:**<br>Bin: >= 1<br>Calling Methodology: Sv Split End, Cnv Read Depth, Str Repeat Expansion, Targeted, Star Allele, Unknown<br>Depth: >= 10<br>50 <= Length <= 10000000<br>MQM: >= 45<br>Quality: HIGH, MODERATE<br>**Polymorphisms filters:**<br>Max all AF: <= 0.03<br>Org db AF, SA-Pathology-nisa-hg38-noise-any-cnv: <= 0.1<br>Org db AF, SA-Pathology-nisa-hg38-noise-any-manta: <= 0.1<br>Org db AF, SA-Pathology-nisa-hg38-noise-any-snv: <= 0.1<br>**Variant type filters:**<br>Variant type: del, dup, ins<br>Variant effect filters:<br>Severity: HIGH, MODERATE<br>**Gene filters:**<br>0: >= 3<br>Candidate genes: True<br>**Zygosity filters:**<br>Proband: HET<br>**Search Filters:**<br>Gene List: NewbornsInSA_AD_ADAR_XL_v1.0
MT-RNR1	**Quality filters:**<br>Allele bias: >= 10<br>Depth: >= 10<br>50 <= Length <= 250000000<br>MQM: >= 45<br>Quality: HIGH, MODERATE

	**Polymorphisms filters:**<br>Max all AF: <= 0.01<br>**Variant type filters:**<br>Variant type: mtdna<br>**Variant effect filters:**<br>Exclusion classification: clinvar, curate, Benign, Likely Benign, 'criteria provided, multiple submitters, no conflicts'<br>Severity: HIGH, MODERATE<br>**Zygosity filters:**<br>Proband: HET, HOM<br>**Other filters:**<br>gene_list_id: 3547
F8	**Quality filters:**<br>Allele bias: >= 20<br>Depth: >= 10<br>MQM: >= 10<br>Quality: HIGH, LOW, MODERATE<br>**Polymorphisms filters:**<br>Max all AF: <= 0.01<br>**Variant effect filters:**<br>Exclusion classification: clinvar, curate, Benign, Likely Benign, 'criteria provided, multiple submitters, no conflicts'<br>Severity: HIGH, LOW, MODERATE, MODIFIER<br>**Zygosity filters:**<br>Proband: HOM<br>**Search Filters:**<br>Gene: F8
GBA1	**Quality filters:**<br>Allele bias: >= 20<br>Depth: >= 10<br>MQM: >= 10<br>Quality: HIGH, LOW, MODERATE<br>**Polymorphisms filters:**<br>Max all AF: <= 0.01<br>**Variant effect filters:**<br>Exclusion classification: clinvar, curate, Benign, Likely Benign, 'criteria provided, multiple submitters, no conflicts'<br>Severity: HIGH, LOW, MODERATE, MODIFIER<br>**Zygosity filters:**<br>Proband: HOM<br>**Search Filters:**<br>Gene: GBA1
CYP21A2	**Quality filters:**<br>Allele bias: >= 20<br>Depth: >= 10<br>MQM: >= 10<br>Quality: HIGH, LOW, MODERATE<br>**Polymorphisms filters:**<br>Max all AF: <= 0.01<br>**Variant effect filters:**

	Exclusion classification: clinvar, curate, Benign, Likely Benign, 'criteria provided, multiple submitters, no conflicts'<br>Severity: HIGH, LOW, MODERATE, MODIFIER<br>**Zygosity filters:**<br>Proband: HOM<br>**Search Filters:**<br>Gene: CYP21A2
SMN1	**Quality filters:**<br>Allele bias: >= 20<br>Depth: >= 10<br>MQM: >= 10<br>Quality: HIGH, LOW, MODERATE<br>**Polymorphisms filters:**<br>Max all AF: <= 0.05<br>Org db AF, SA-Pathology-nisa-hg38-noise-any-cnv: <= 0.1<br>Org db AF, SA-Pathology-nisa-hg38-noise-any-manta: <= 0.1<br>Org db AF, SA-Pathology-nisa-hg38-noise-any-snv: <= 0.1<br>**Variant effect filters:**<br>Exclusion classification: clinvar, curate, Benign, Likely Benign, 'criteria provided, multiple submitters, no conflicts'<br>Severity: HIGH, LOW, MODERATE, MODIFIER<br>**Zygosity filters:**<br>Proband: HOM<br>**Search Filters:**<br>Gene: SMN1

Supplementary Table 6. Filtering parameters used to flag a variant 'For review' by the post-processing automation script.

Preset	Parameters
**Most likely by *AI***	<u>For recessive only</u> genes (NISA domain Gene List ID = 4805):<br>o Zygosity = HOM **AND** variant is:<br>• Known ClinVar Pathogenic / Likely Pathogenic (ClinSig = 4,5) **OR**<br>• Impact = High **OR**<br>• Impact = Moderate AND Splice Prediction = High, Moderate **OR**<br>• Impact = Moderate AND *In Silico* Prediction = Damaging<br><br>o Zygosity for variant(s) in the same gene = HET, HET **AND both variants with** any of the following:<br>• Known ClinVar Pathogenic / Likely Pathogenic (ClinSig = 4,5) **OR**<br>• Impact = High **OR**<br>• Impact = Moderate AND Splice Prediction = High, Moderate **OR**<br>• Impact = Moderate AND *In Silico* Prediction = High, Moderate
	Dominant, X-linked and AD/AR genes (NISA domain Gene List ID = 3544), and *MT-RNR1* (NISA domain Gene List ID = 3547):<br>o Zygosity = HET, HOM **AND** any of the following:<br>• Known ClinVar Pathogenic / Likely Pathogenic (ClinSig = 4,5) **OR**<br>• Impact = High **OR**<br>• Impact = Moderate AND Splice Prediction = High, Moderate **OR**<br>• Impact = Moderate AND Prediction = High, Moderate
**NISA – Hom**	Recessive genes (NISA domain Gene List ID = 4621):<br>• ClinVar Pathogenic / Likley Pathogenic (ClinSig = 4,5) **OR**<br>• Impact = High **OR**<br>• Impact = Moderate AND Splice Prediction = High, Moderate **OR**<br>• Impact = Moderate AND *In Silico* Prediction = Damaging
**NISA – C.Het**	Recessive genes (NISA domain Gene List ID = 4621):<br>o Zygosity for variant(s) in the same gene = HET, HET **AND both variants with** any of the following:<br>• Known ClinVar Pathogenic / Likely Pathogenic (ClinSig = 4,5) **OR**<br>• Impact = High **OR**<br>• Impact = Moderate AND Splice Prediction = High, Moderate **OR**Impact = Moderate AND *In Silico* Prediction = High,

	Moderate
**NISA – AD**	Dominant genes (NISA domain Gene List ID = 3543):<br>• Known ClinVar Pathogenic/Likely Pathogenic (ClinSig = 4,5) **OR**<br>• Impact = High **OR**<br>• Impact = Moderate AND Splice Prediction = High, Moderate **OR**<br>• Impact = Moderate AND Prediction = Damaging
**NISA – *De novo***	NISA domain Gene List ID = 3544:<br>• Known ClinVar Pathogenic/Likely Pathogenic (ClinSig = 4,5) **OR**<br>• Impact = High **OR**<br>• Impact = Moderate AND Splice Prediction = High, Moderate **OR**<br>• Impact = Moderate AND Prediction = Damaging
**NISA - XL**	NISA domain Gene List ID = 3548, 3549:<br>• Known ClinVar Pathogenic/Likely Pathogenic (ClinSig = 4,5) **OR**<br>• Impact = High **OR**<br>• Impact = Moderate AND Splice Prediction = High, Moderate **OR**<br>• Impact = Moderate AND Prediction = Damaging
**NISA – ClinVar Hom / C.het / AD / XL**	Variant present = Yes?<br>• Known ClinVar Pathogenic/Likely Pathogenic (ClinSig = 4,5)
**NISA – MtDNA**	NISA domain Gene List ID: 3547<br>• Known ClinVar Pathogenic/Likely Pathogenic (ClinSig = 4,5) **OR**<br>• MitoMap Known

* Variant Impact High or Moderate effect as defined by Ensembl's VEP
